# Effect of Carbonic Acid on the Gravid Uterus

**Published:** 1857-01

**Authors:** 


### *Effect of Carbonic Acid on the Gravid Uterus.*

The *Union Médicale*, for August 12, contains an account of a case in which carbonic acid gas, injected into the vagina, was successfully employed to effect premature delivery. The gas was generated into a glass vessel, by means of the action of acetic acid on the bicarbonate of soda, and conducted into the vagina by means of an elastic tube. A glass speculum was introduced into the vagina, and in order to prevent the carbonic acid from escaping too freely, the tube was passed through a cork which closed the external opening of the speculum. The injection of the acid was followed by a disagreeable pricking sensation in the vagina. After the third application, some pain was felt in the umbilical region, and the cervix uteri became softened. Six applications were made, of from twenty to thirty minutes each, one at night and one in the morning; after the last one, uterine contractions came on, and delivery followed. The result of this case is very satisfactory, so far as it goes, and is worthy of trial in cases where it becomes necessary to procure premature delivery, especially as it occasions no harm either to mother or child, and at the worst only causes the delay of a few days.—*Boston Med. and Surg. Journal.*

